# Systematic underestimation of the age of selected alleles

**DOI:** 10.3389/fgene.2012.00165

**Published:** 2012-08-31

**Authors:** Joanna L. Kelley

**Affiliations:** Department of Genetics, Stanford UniversityStanford, CA, USA

**Keywords:** haplotype length, selected allele, selective sweep, allele age, time to most recent common ancestor, tMRCA

## Abstract

A common interpretation of genome-wide selection scans is that the dispersal of anatomically modern humans out of Africa and into diverse environments led to a number of genetic adaptations. If so, patterns of polymorphism from non-African individuals should show the signature of adaptations dating to 40,000–100,000 Kya, coinciding with the main exodus from Africa. However, scans of polymorphism data from a few populations have yielded conflicting results about the chronology of local, population-specific adaptations. In particular, a number of papers report very recent ages for selected alleles in humans, which postdate the development of agriculture 10 Kya, and suggest that adaptive differences among human populations are much more recent. I present an analysis of simulations suggesting a downward bias in methods commonly used to estimate the age of selected alleles. These findings indicate that an estimate of a time to the most recent common ancestor (tMRCA) obtained using standard methods (used as a proxy for the age of an allele) of less than 10 Kya is consistent with an allele that actually became selected before the onset of agriculture and potentially as early as 50 Kya. These findings suggest that the genomic scans for selection may be consistent with selective pressures tied to the Out of Africa expansion of modern human populations.

## Introduction

We can learn about how and when selection has acted on a genomic region by using genetic variation data within a species to date the age of selected alleles. For example, in *Drosophila melanogaster*, researchers can identify which loci have responded to range expansion into temperate climates (David and Capy, 1988) or in domestic crops, whether the timing of selection coincides with the advent of agriculture or subsequent crop improvement (Purugganan and Fuller, 2009). Precisely dating the onset of selective pressure also has implications for our understanding of human evolution. Humans migrated out of Africa approximately 100,000 years ago and subsequently colonized the globe (Jobling et al., 2004). Accurately estimating the age of selected alleles helps to frame the anthropological context of selection and provides hints as to the nature of selective pressures.

**Figure 1:**
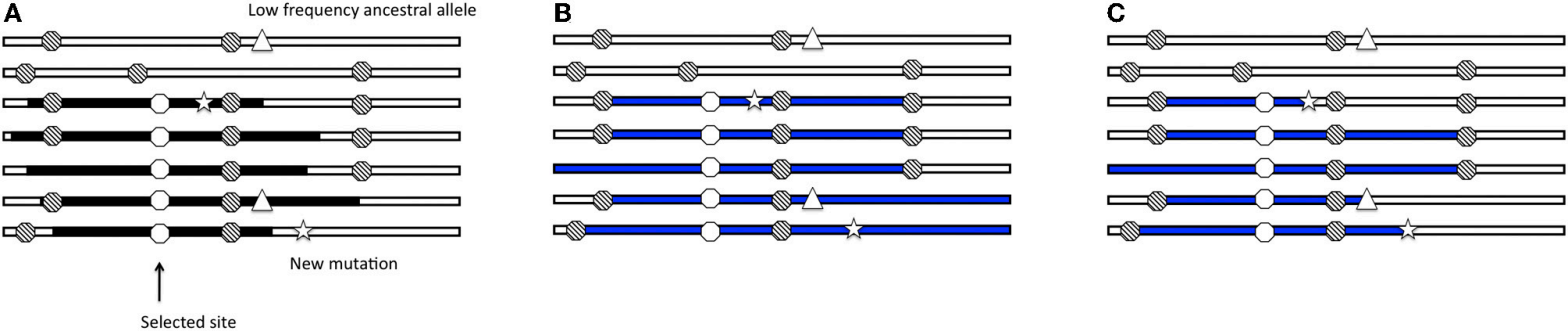
**Selective sweep with recombination.** Cartoon of seven chromosomal regions with neutral, segregating alleles (striped dots); the black segments are the chromosomal segments carrying the selected allele (white dot). **(A)** The region surrounding the incomplete selective sweep harbors a mix of long haplotypes carrying the selected allele (black segments) and ancestral haplotypes (white segments). There are also low frequency ancestral alleles (triangle) and new mutations (star) in the region. **(B)** Inferred haplotype lengths (blue) by the haplotype-based method applied to ascertained SNP data. **(C)** Inferred haplotype lengths (blue) by the haplotype-based method applied to full-resequencing data.

Over the past few years, researchers have used genome-wide polymorphism and divergence data to identify hundreds of putatively selected genes in the human genome (Sabeti et al., 2002; Clark et al., 2003; Akey et al., 2004; Bustamante et al., 2005; Carlson et al., 2005; International HapMap Consortium, 2005; Nielsen et al., 2005; Kelley et al., 2006; Voight et al., 2006; Wang et al., 2006). These genome-wide scans are based on statistics that summarize variation data. The idea behind these statistics is that the increase in frequency of a beneficial allele in the population decreases nucleotide variation in a closely linked region of the genome (in a “selective sweep”). After fixation, new (rare) mutations arise, leading to a quantifiable shift in the site-frequency spectrum as compared to neutral regions. Additionally, recombination events between chromosomes carrying the selected allele and chromosomes without the selected allele also contribute to the shift in the site-frequency spectrum as high frequency and low frequency derived variants are present in the region surrounding the selected allele (Smith and Haigh, 1974). An incomplete sweep (where the favored allele does not reach fixation) does not lead to a dramatic deviation from the neutral expectation of the site-frequency spectrum (Simonsen et al., 1995). However, the region surrounding the site of an incomplete selective sweep will harbor a mix of long stretches of identical haplotypes carrying the selected allele and ancestral haplotypes of varying lengths, affecting patterns of linkage disequilibrium in the region (Figure 1A). Thus, considering different aspects of the data allows researchers to identify selective events that occurred at different times in human history (Sabeti et al., 2006).

In humans, the signature of adaptation that occurred since the last Ice Age or during the Out of Africa migration should still be identifiable from sampling individuals today. Population-genetic theory predicts that a selective sweep takes approximately 2 ln(2 Ns)/s generations to complete, where N is the effective population size and s, the selection coefficient, is the advantage of the selected allele (Hermisson and Pennings, 2005). Therefore, the time to completion of a selective sweep is highly dependent on the strength of selection. Given the predicted long-term human effective population size of 10,000 (Takahata, 1993), 25 years per generation, and realistic selection coefficients (*s* < 5%) (Bersaglieri et al., 2004), we expect that selective sweeps occurring after the human migration from Africa will typically be incomplete. Therefore, selected alleles currently at intermediate frequency are either recent and strong or old and more weakly selected.

As increasing numbers of candidate selected loci are identified, the focus is shifting from constructing catalogues of putatively selected loci to understanding why the loci have been positively selected, the functional relevance of the selected allele, and the timing of the selective pressure. In particular, an important question for our understanding of recent human evolution is the extent to which selective signatures detected in the human genome reflect strong, post-agricultural pressures as opposed to older, perhaps more weakly selected events that occurred since the expansion of modern humans out of Africa (Sabeti et al., 2002; Clark et al., 2003; Akey et al., 2004; Bustamante et al., 2005; Carlson et al., 2005; International HapMap Consortium, 2005; Nielsen et al., 2005; Kelley et al., 2006; Voight et al., 2006; Wang et al., 2006). To distinguish between these hypotheses, it is helpful to date the onset of the selective pressure.

**Figure 2:**
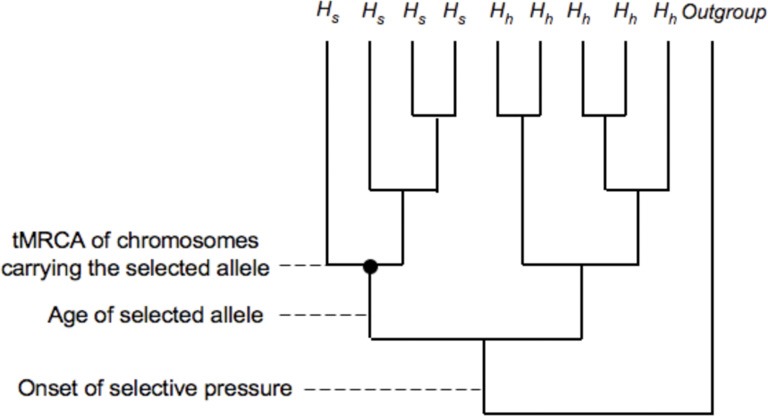
**Selection acting on a new mutation.** Comparison between time to most recent common ancestor (tMRCA) for a set of selected alleles and the age of the selected allele. *H*_*s*_ represents haplotypes with the selected allele and *H*_*h*_ represents neutral haplotypes.

While primarily interested in timing the onset of selection, researchers use the age of the selected allele, or the time to the most recent common ancestor (tMRCA), of the haplotypes carrying the selected allele, as proxies for the onset of selective pressures, since the time between the onset of a selective pressure and the appearance of a relevant selected mutation in the population is unknown. Assuming that selection acts on a new mutation, the tMRCA will be more recent than the onset of selection (Figure 2) and is likely to be within two-fold of the age of the allele (Teshima and Przeworski, 2006). Methods have been developed to estimate the age of rare alleles, high frequency alleles or alleles that are fixed along the human lineage (Slatkin and Rannala, 2000; Thomson et al., 2000; Przeworski, 2003).

Several current studies have estimated the age of putatively selected alleles in the human genome dating to within the most recent 10 Kya (Voight et al., 2006; Hawks et al., 2007). These findings have been interpreted as evidence for adaptation in the human genome being recent and falling squarely within the agricultural phase of human population history. However, another study using a similar method estimates that selective events occurred between 40,000 and 10,000 years ago (Wang et al., 2006). The context in which selected mutations arose in the human genome and the timing of those mutations sheds light on what selective events were most influential in shaping extant variation.

Here I consider the performance of one main type of estimator that has been applied to learn about the age of a selected allele at intermediate frequency—the outcome that would be expected for strong, relatively recent selection in humans. The methods implemented by Voight and colleagues (2006) and Wang et al. (2006) rely on *haplotype decay* to estimate the ages of the alleles with evidence for positive selection. The methods are based on simple models for the probability of observing two chromosomes that are identical by descent at a given recombination distance from the selected site, given that the two chromosomes share a common ancestor at some time point ago. The method uses this relationship to estimate the tMRCA.

## Methods

One common approach was taken by Voight and colleagues (2006), who considered the probability that the two chromosomes are homozygous a given recombination distance from the putatively selected site:

$$\text{Pr}[\text{Homoz}]={\text{e}}^{-\text{2rgT}}$$

where T is the tMRCA in generations, g is the generation time and r is the recombination distance to which the identity between the two chromosomes extends. Often, in applications to humans, a generation time of 25 years is used to convert generation time estimates to year estimates. The method makes simplifying assumptions to obtain age estimates–for example, that the population is of constant size and panmictic. The method assumes a star shaped phylogeny at the selected site. The method is implemented by determining the recombination distance from the selected allele to the nearest single nucleotide polymorphism (SNP), which is assumed to be the nearest recombination event from the selected background to the ancestral background (Figure 1B). For each chromosome with the selected allele, the recombination distance from the selected site to the nearest recombination event SNP on either side of the selected allele is recorded. In the implementation, when 75% of chromosomes have recombined off of the selected haplotype (Pr[Homoz] = 0.25), the recombination distance (*r*) is input into the formula above. The method assumptions are unlikely to be realistic. Thus, there are a number of reasons to suspect that the method may not be robust to model assumptions. To date, there has been no assessment of the method's reliability.

Here, I evaluated the performance of haplotype-based dating methods. The methods were applied to simulated data generated under a wide range of parameters. To generate the simulated data, I used the program mssel, a modified version of ms that allows for selection at one site (kindly provided by Richard Hudson). mssel uses a coalescent approach to generate samples from a neutrally-evolving region linked to a site at which an allele is under selection (Kaplan et al., 1989; Braverman et al., 1995). The user specifies the trajectory of the selected allele, including the present-day frequency of the allele in the population. Phase is assumed to be known.

The demographic models considered included constant population size with *N*_*e*_ = 10,000, as well as bottleneck and two population growth models, a recent and an ancient expansion, thought to describe the demographic history of particular human populations since anatomically modern humans migrated out of Africa (Ramachandran et al., 2005; Schaffner et al., 2005; Voight et al., 2005). The bottleneck model is similar to that inferred for European population history, with a present day effective population size of 10,000, a bottleneck population size of 520 that started 1400 generations ago and lasted 200 generations. The ancient expansion model is a doubling of population size to 20,000 chromosomes, 7440 generations ago. The recent expansion demography model has the population size doubling to 20,000 chromosomes, 1000 generations ago.

The trajectory of the selected allele was simulated under the different parameters and demographies using a forward time simulation of the Wright-Fisher model, implemented in R. The trajectories were input into mssel with the corresponding demographic model, assuming a uniform recombination rate of 10^−8^ and mutation rate of 10^−8^.

From these simulations, I evaluate the bias of tMRCA estimates for the method by considering the log_2_ estimated value over the true value because a value of 1.0 or −1.0 corresponds to a two-fold over or under-estimate, respectively. I also summarize the root mean squared error (RMSE) as a measure of accuracy. The method was applied as in Voight et al. (2006), with a cut-off of Pr[Homoz] = 0.25. As in haplotype-based dating methods, I assumed haplotypes were known, as haploid data was simulated, but the recombination breakpoint locations were not known. The relationship between the tMRCA and the age of the selected allele depends in part on the dominance coefficient of the selected allele (Teshima and Przeworski, 2006). I therefore considered a range of dominance coefficients and frequencies of the selected allele in the population to gauge how these parameters influences the tMRCA estimates.

## Results

**Figure 3:**
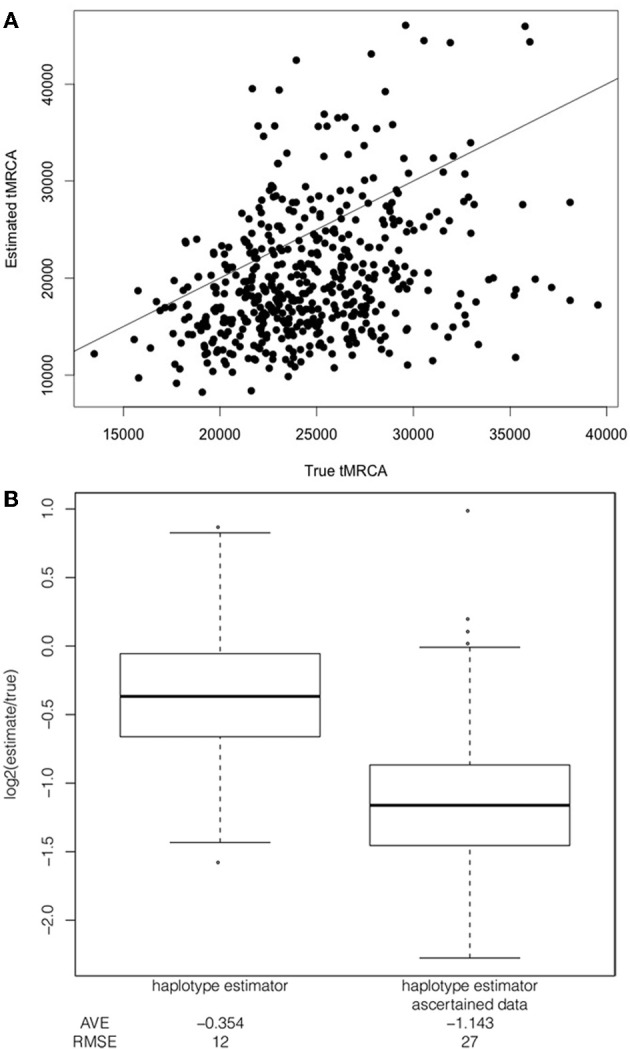
**Comparison of bias in estimator applied to full sequence data and ascertained data.** Comparison of the true to estimated tMRCA **(A)** Example of direct comparison between true tMRCA and estimated tMRCA when applied to full sequence data simulated under a constant population size, uniform recombination rate, a selection coefficient of 1%, additive dominance and with the selected allele at a frequency of 50% in the population and the sample, with line x = y for reference. **(B)** Boxplot of log_2_ ratio of the tMRCA estimate to the true tMRCA for the two estimators (applied to full sequence data and ascertained data) under a constant population size, uniform recombination rate, a selection coefficient of 1%, additive dominance and with the selected allele at a frequency of 50% in the population and the sample. A value of 1.0 or −1.0 corresponds to a two-fold over or under-estimate, respectively. The root mean squared error (RMSE) and average log_2_ ratio (AVE) is included below each boxplot.

Under a constant demographic model with a selection coefficient of 1%, additive dominance model and the frequency of the selected allele at 50% in the population and the sample, the mean tMRCA is 24,579 years. In turn, the estimated tMRCAs are downward biased with an average log_2_ estimate over truth of −0.354 [mean estimated tMRCA is 19,914 years and a RMSE of 12 (Figure 3)]. Thus, the estimated ages are downwards biased using the haplotype decay method.

To date, the haplotype decay methods (as well as other dating methods) have been applied to genotype data that have a number of ascertainment biases. I modeled SNP ascertainment by randomly selecting five of the simulated chromosomes for SNP discovery and subsequently using only those sites segregating among the discovery panel. This ascertainment scheme biases in favor of moderate to higher frequency alleles, and is simpler than but along the lines of ascertainment schemes used to discover SNPs for HapMap and Perlegen projects (Clark et al., 2005). An exploration of the ascertainment of SNPs in the simulated data revealed that the haplotype decay estimator is particularly sensitive to the ascertainment scheme and more biased when only intermediate and high frequency SNPs are used to calculate the estimate. For example, in the case of the constant demographic model, selection coefficient of 1%, additive dominance model and the frequency of the selected allele at 50% in the population and the sample, the haplotype decay method is more biased when applied to genotype data than when applied to full sequence data (Figure 3). Using full sequence data appears to decrease the bias as compared to genotyping data. We postulate that this is due to several factors which act to affect the estimated age: (1) true recombinations that occur between haplotypes carrying the selected allele and linked sites are unseen using ascertained SNP markers and (2) low frequency ancestral alleles are unseen using ascertained SNP markers and (3) newly arisen mutations on some haplotpyes which are observed by analyzing full sequence data but which are erroneously counted as recombination events (Figure 1C).

**Figure 4:**
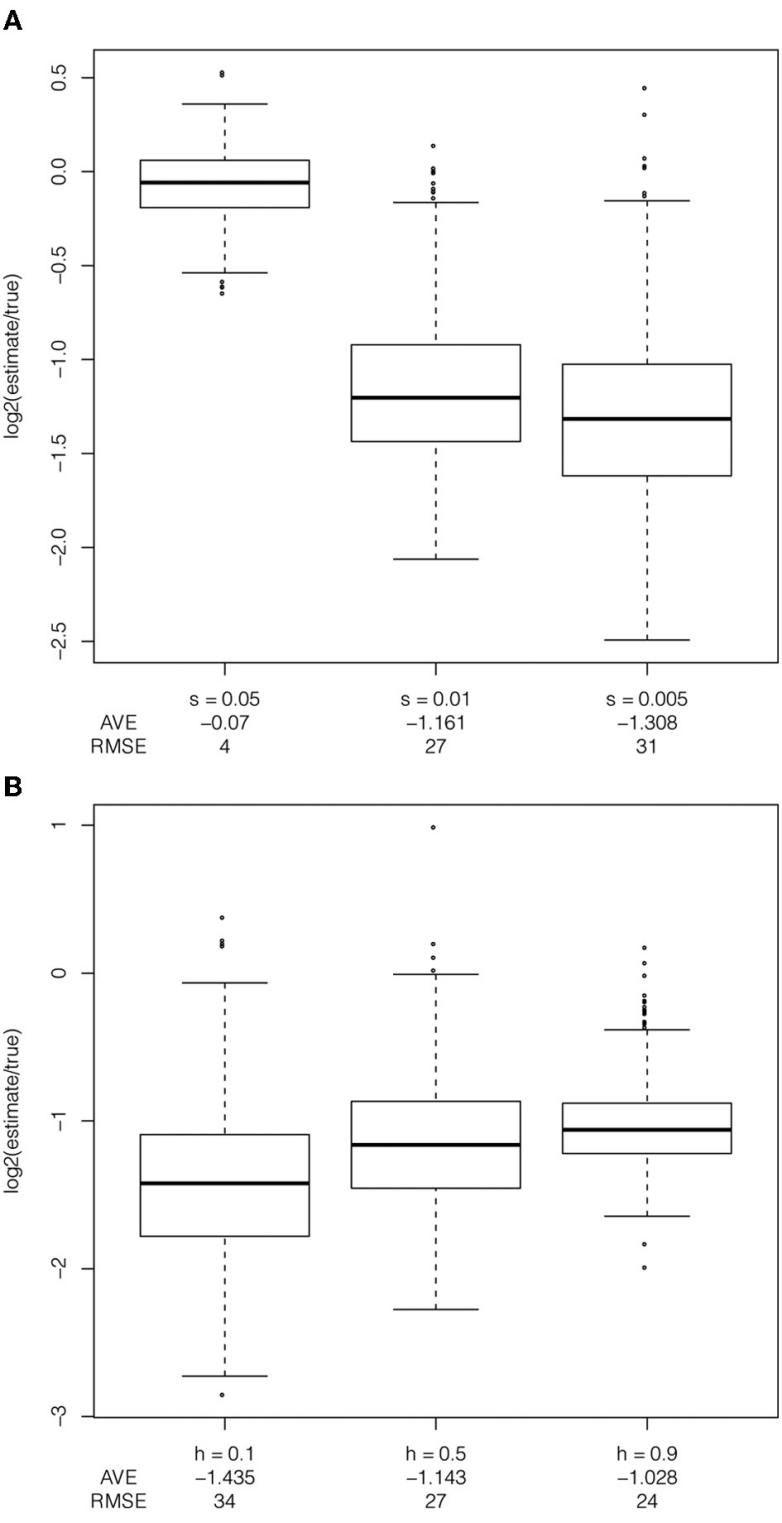
**Exploration of selection coefficients and dominance coefficients.** Exploration of **(A)** selection coefficient (*s* = 5%, 1% and 0.5%), under a recent expansion demographic model, with ascertainment bias, with a additive dominance model and the frequency of the selected allele at 50% in the population, and **(B)** dominance coefficient, under a constant demographic model, with ascertainment bias, selection coefficient of 1% and the frequency of the selected allele at 50% in the population.

To evaluate the effect of selection and dominance coefficients on the estimator, I simulated data varying one parameter and holding the demographic model and either the selection coefficient or dominance coefficient constant. Bias is more pronounced with decreasing selection coefficient (Figure 4A). With high selection coefficients (*s* = 5%), this bias is reduced and the true tMRCA is closer to the true age. These are the only scenarios in which the true age of the selected allele was in the most recent 10,000 years. With larger selection coefficients, the model assumption of a star-shaped phylogeny is likely appropriate, whereas this is often violated in the cases of smaller selection coefficients. The smaller, and perhaps more realistic, selection coefficients lead to a larger bias in the haplotype decay method. The effective population size times the selection coefficient (*N*_*e*_*s*) is a relevant parameter for determining the trajectory of the selected allele, while I did not directly estimate the effect of changing effective population size on the estimator, these simulations suggest that increasing *N*_*e*_ with a fixed selection coefficient will decrease the bias in the estimation of the tMRCA (Figure 4A).

The dominance coefficient of the selected allele also biases the haplotype-based estimator. Under a recessive model, conditional on reaching an intermediate frequency in the sample, the selected allele spends more time at low frequency as compared to additive or dominant models. This leads to both an increase in the distance between the true age of the selected allele and the true tMRCA and the distance between the estimated tMRCA and the true tMRCA (Figure 4B, average log_2_ estimate/true less than −1.4 for *h* = 0.1). As the dominance coefficient increases, there is less bias in the estimator. However, the estimator is biased regardless of the selection coefficient or dominance coefficient.

**Figure 5:**
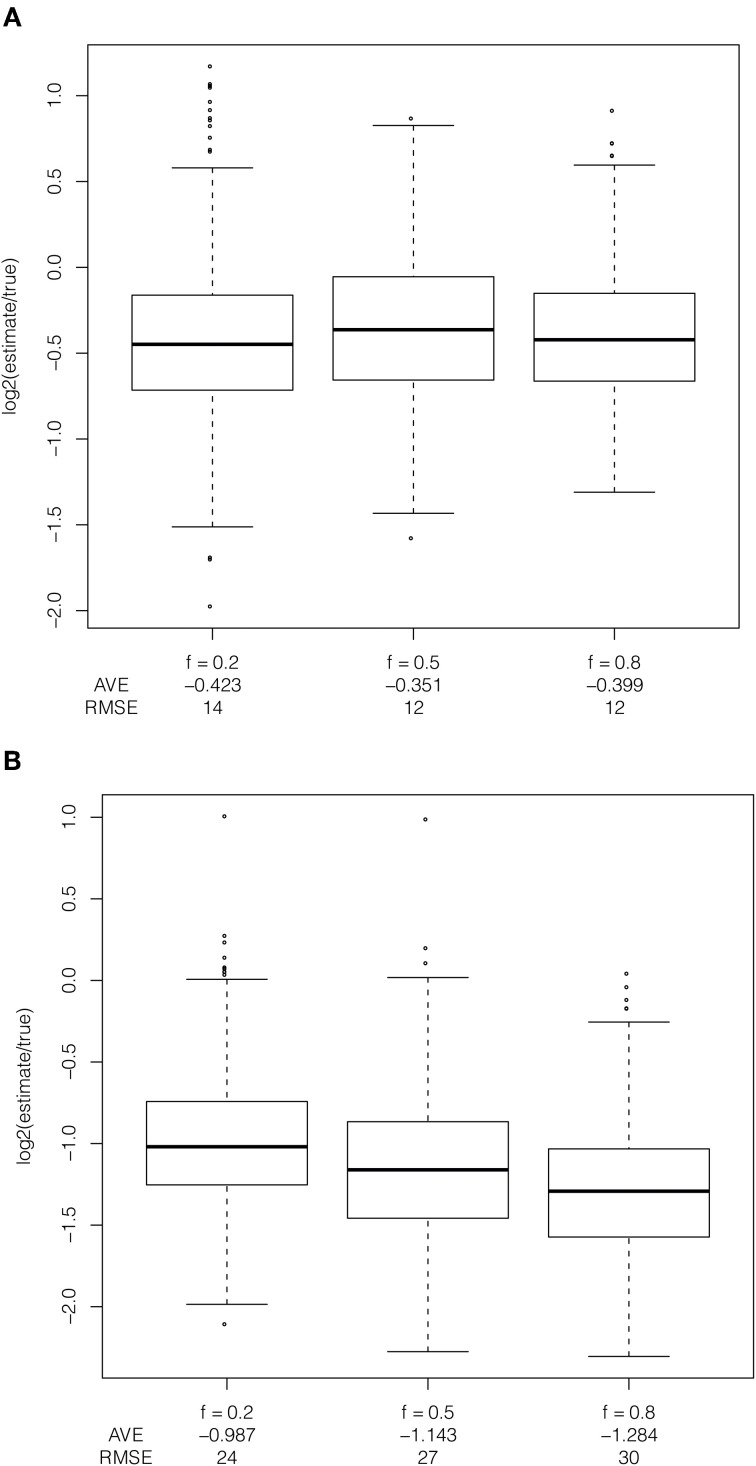
**Effect of frequency of the selected allele in the population.** Comparison of bias in estimator due to frequency of the selected allele applied to **(A)** full resequence data and **(B)** ascertained data with constant population size, uniform recombination rate, selection coefficient of 1%, additive dominance and with the selected allele at a frequency of 20%, 50%, and 80% in the population and 50% in the sample.

The frequency of the selected allele in the population leads to bias in the estimator, only when applied to ascertained data (Figure 5). When the selected allele is at high frequency in the population (frequency of 80% in the population and 50% in the sample), some recombination events occur between haplotypes carrying the selected allele and are more likely to be unobserved. When the selected allele is at low frequency in the population recombination events move the selected haplotype onto the non-selected background, which has SNPs tagging it due to ascertainment of common alleles. The frequency of the selected allele has little effect on bias when the estimator is applied to full sequence data because new mutations are incorrectly counted as recombination events because the model does not permit new mutations. A new mutation breaks the length of the inferred shared haplotype in the implementation of the method (see Figure 1C). This is particularly relevant for human data because the mutation rate and recombination rate are of the same order of magnitude.

**Figure 6:**
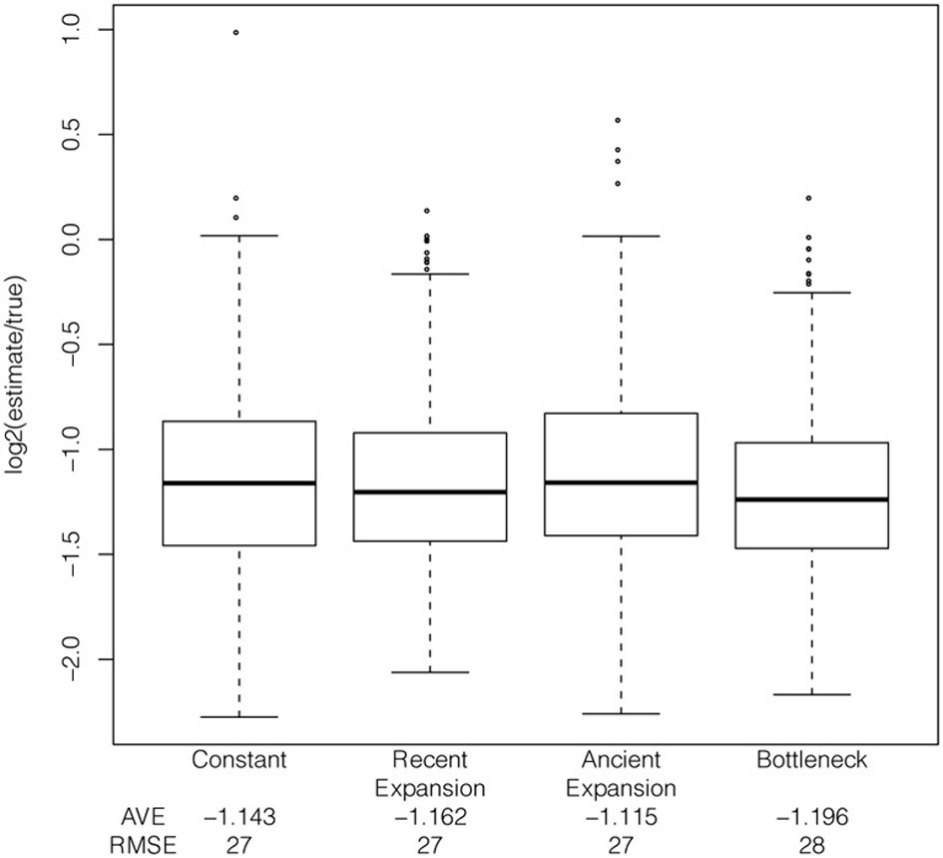
**Effect of demography on tMRCA estimates.** Comparison of bias under four demographic models, using simulated ascertained data with a selection coefficient of 1%, additive model, and the frequency of the selected allele at 50% in the population.

Simulating the trajectory of the selected allele and the haplotypes in mssel under the demographic models described above reveals that the extent of bias is relatively consistent across several demographic models, but slightly more pronounced under some bottleneck models (*s* = 1%, additive model, Figure 6). The bias in the estimator is relatively consistent across different demographic models for selection coefficients of 1% and 5%; however, as the selection coefficient decreases, *s* = 0.5%, the bias in the estimator varies between demographic models. Additionally, the relationship between the true tMRCA and allele age varies between demographic models. The estimator is biased but relatively precise; therefore, while absolute estimates are unreliable, the method could be used to order selective events.

## Discussion

These simulations suggest that the estimated ages that researchers have reported for selected alleles are likely to be quite a bit younger than the true age of the selected allele. As described, a number of reasons lead to this discrepancy, including ascertainment bias and the effects of the selective and dominance coefficients. The haplotype-based estimated tMRCA is about half the true tMRCA, especially when applied to ascertained genotype data. In scenarios with low selection coefficients or recessive alleles, the true tMRCA is about half the age of the selected allele. Overall, the simulations suggest that the tMRCA is at least about half the age of the selected allele, which is itself younger than the onset of selective pressure. Moreover, there is an additional consideration in interpreting the results: By selecting loci that are identified as having strong statistical evidence of having been subject to recent positive selection, there is an implicit conditioning on them having risen to high frequency quickly and therefore on having a more recent tMRCA than an *average* selected allele responding to the same selective pressures, a form of the winner's curse (as in Hawks et al., 2007, Figure 1). An additional consideration posed by these results is that it is important to consider the predicted strength of selection, dominance coefficient and the ascertainment scheme to choose the appropriate estimator for the data. The results suggest that in the case with very strong selection (*s* = 5%) or very large population size the haplotype-based estimator does well at estimating the true tMRCA.

As humans migrated out of Africa, they encountered new environments and were challenged with new infectious agents. Additionally, the rise and spread of agriculture had a profound impact on human way of life. Accurately estimating the age of selected alleles shapes our understanding of selective events during recent human history. We can reconcile the times with potential selective forces, for example agriculture or pathogens, which have contributed to the adaptive evolution of humans. The finding, that the selected alleles that have been dated using these methods may be much older than previously thought, suggests that adaptive events affecting extant variation in the human genome date to much earlier than the onset of agriculture, and are likely due to pressures encountered when populations were colonizing the globe.

Accurately determining the timing of selective events and accurately quantifying biases in existing methods, when considered together with information about the geographic distribution of allele frequencies, should be of great use in interpreting the signals of selection and understanding how selected alleles have swept through populations. However, to reliably interpret the results, simulations are needed—of hard sweeps, such as modeled here, as well as of more complex selective scenarios, such as soft sweeps and selection on standing variation (Coop et al., 2009; Pritchard et al., 2010), which are likely important contributors to patterns of variation in the genome (Hernandez et al., 2011).

### Conflict of interest statement

The author declares that the research was conducted in the absence of any commercial or financial relationships that could be construed as a potential conflict of interest.
